# Model construction for estimating potential vulnerability of Japanese soils to cadmium pollution based on intact soil properties

**DOI:** 10.1371/journal.pone.0218377

**Published:** 2019-06-14

**Authors:** Kyoko Ono, Tetsuo Yasutaka, Takehiko I. Hayashi, Masashi Kamo, Yuichi Iwasaki, Taizo Nakamori, Yoshikazu Fujii, Takafumi Kamitani

**Affiliations:** 1 Research Institute of Science for Safety and Sustainability, National Institute of Advanced Industrial Science and Technology, Tsukuba, Ibaraki, Japan; 2 Research Institute for Geo-Resources and Environment, National Institute of Advanced Industrial Science and Technology, Tsukuba, Ibaraki, Japan; 3 National Institute for Environmental Studies, Tsukuba, Ibaraki, Japan; 4 Graduate School of Environment and Information Sciences, Yokohama National University, Yokohama, Kanagawa, Japan; 5 Environmental Education Center, University of Human Environments, Okazaki, Aichi, Japan; 6 Shizuoka Institute of Environment and Hygiene, Shizuoka, Shizuoka, Japan; The University of Texas at El Paso, UNITED STATES

## Abstract

Prediction of heavy metal bioavailability in intact soil is important to manage soil pollution risks. We developed a regression model for representative Japanese soils to judge their potential vulnerability to cadmium (Cd) pollution. We added four rates of Cd to 17 sample soils to mimic artificial contamination. After aging the contaminated soils, we measured Cd’s bioavailability using the diffusive gradients in thin-films (DGT) technique. We then evaluated the relationships between bioavailability of Cd ([Cd_DGT_]) and intact soil properties by statistical analyses. Cation exchange capacity (CEC) and pH emerged as significant factors to explain the cadmium bioavailability in Japanese soils. Specifically, lower CEC and lower pH were associated with higher [Cd_DGT_], which poses a higher risk for soil ecosystems. The correlation between pH and [Cd_DGT_] had a high dependence on [Cd_Add_], whereas that for CEC did not. Regression analysis also showed that the interaction between intact soil pH and spiked concentration ([Cd_Add_]) had a significant contribution to [Cd_DGT_]. The regression model developed was rationally supported by a biotic ligand model. This simplified but realistic model would be useful in estimating the vulnerability of representative Japanese soils and determining the risk for Japanese soils in relation to Cd contamination.

## Introduction

Cadmium (Cd) is a major contaminant of soils in Japan and many other countries. Adequately evaluating the impacts of heavy metals such as Cd in soils is important for ecological risk assessments for soils, facilitating proactive decision making for regulation of soil ecosystems. Some researchers have devised a method to derive “critical limits” or a “critical load” to soil based on potential bioavailability in order to draw a vulnerability map of European soils [1–3]. They categorized soil types vulnerable to contamination according to the extent of metal bioavailability, and thus identified soil ecosystems with higher ecological risks. In Japan, however, there is very little information available for setting a data-based contamination criterion, and there is no established soil vulnerability map to show higher-risk area(s).

Metal impacts on soils depend on the bioavailability of the metals in the soil, not the total content [4–6]; thus, understanding bioavailability in soil and developing models to predict it from soil properties are critically important. For example, the metals responsible for toxicity are known to be in the free-ion form. Metal speciation is a key to understanding bioavailability, but analytical procedures for speciation are often labor intensive. The sequential extraction method has been used to evaluate the bioavailability of metals in soils by dividing the metals into different fractions [7–9]. This method can provide useful information on the behavior, bioavailability, and toxicity of metals by evaluating chemical speciation [10]. However, the sequential extraction method has a limitation in that the extracted fraction does not always correlate with the degree of bioavailability.

The so-called “diffusive gradients in thin-films” (DGT) method was developed as an alternative and simpler method to evaluate the bioavailability of heavy metals. It measures the amount of metals bound to ion exchange resin [11]. The DGT technique enables the direct measurement of metal-labile fractions by mimicking the gills of fish or plant roots. Because it enables the measurement of labile fractions directly with a simple manipulation [12], its applicability to soil has been tested by comparing DGT-measured concentrations of metals with those in soil solution [13], for uptake in plants [14–19], and for uptake in snails [20]. The DGT method gave a higher correlation between the cadmium (Cd) concentrations ([Cd]) in roots and grains of rice and [Cd] in soil than did the soil solution, acetic acid extraction, and CaCl_2_ extraction methods [21]. It also gave a good correlation between [Cd] in soil and [Cd] in earthworms [22] and adverse effects observed in earthworms [23]. Thus, the DGT method is now used to evaluate the bioavailability of heavy metals in soils.

It is important to understand the series of processes involved in converting total metal concentration into bioavailability such as internal dose of metals in biota. It is well known that the following factors influence the bioavailability of soil: history of contamination (aging) [24] and soil properties [25], including pH [26], CEC, organic matter content, and clay content. It is a challenging work to describe the physico-chemical properties that are incorporated into affinity constants in metal kinetics in an environmental medium, and to understand absorption mechanisms on soil ligands with metals and other cations. Most studies so far (e.g. [27,28]) have attempted to understand the roles of soil physico-chemical properties in the link between environmental concentration of metals and the internal dose of metals in biota. However, surprisingly few studies have evaluated the relationships between DGT-estimated metal bioavailability and soil physico-chemical properties such as cation-exchange capacity (CEC) and pH [17,29]. Furthermore, a model using parameters of intact (i.e. uncontaminated) soil properties is lacking.

The aims of this study were to evaluate the relationships between added (i.e. artificially contaminated) Cd concentration and Cd bioavailability measured by the DGT method as a function of physicochemical properties such as cation exchange capacity (CEC) and pH, and to propose a linear regression model for soil bioavailability that is reasonably applicable to Japanese soils for potential vulnerability assessment. To those ends, we evaluated the relationships between soil properties and bioavailability of Cd by a biotic ligand model.

## Materials and methods

### Collection, preparation, and analysis of soil samples

We collected 16 soil samples throughout Japan: 5 sandy soils, 4 andosols, 3 brown forest soils and 4 cohesive soils, and we prepared 1 artificial soil mixed to an OECD standard [30]. Most of the soil samples were provided from research fields of universities or research institutes and purchased from the market (see S1 Table for more details). The permission was obtained to collect all the soil samples and we confirm that those soils did not involve endangered or protected species.

The sample preparation methods have been described elsewhere in detail [31]. Briefly, each sample (>1000 g) was air dried and sieved by a 2-mm sieve before analysis. The soil type was identified according to the classification of cultivated soils in Japan [32]. The particle size fraction was analyzed according to JIS A1204 [33]. Samples were fractioned through a series of sieves [33]; then, fractions of ≥75 μm were weighed, and fractions of <75 μm were determined by a sedimentation method using a hydrometer. Cation exchange capacity (CEC) was determined by the Schollenberger method [34]. Specific surface area (AREA) was measured by a gas adsorption method (ASAP, 2020; Shimadzu corporation, Kyoto, JAPAN). Water-holding capacity (WHC) was measured according to ISO 11268–1 [35]. Soil pH was measured in a soil-to-water ratio of 1:1 (w/v) by a portable pH meter (WM-32EP DKK-TOA corporation). Total carbon content (total C) was measured with an elemental analyzer (Thermo Electron Corporation, FLASH EA 1112 Series). Ignition loss was determined by the JIS A 1226 [36] test method. The soil properties are summarized in Table 1. Since properties of OECD soil are very similar to those of cohesive soils, OECD soil was treated as one of the cohesive soils.

**Table 1:** Physical and chemical properties of sample soils.

No.	Sampling location	Sand (%)	Silt (%)	Clay (%)	CEC (cmol/kg)	AREA (m^2^/g)	WHC (%)	pH (H_2_O)	Total C (%)	Ignition loss (%)	Soil type
S-1	Toyama	87.8	7.5	4.7	1.7	14	23	7.08	0.02	0.6	Sandy soil
S-2	Shizuoka	95.0	1.2	3.7	0.6	23	43	6.32	0.10	0.3	
S-3	Gifu	63.3	15.5	10.1	4.0	33	31	5.69	2.09	10.1	
S-4	Nagano	92.9	5.0	2.1	1.9	31	27	6.20	0.01	1.2	
S-5	Yamaguchi	99.9	0.1	0.0	0.5	28	28	6.60	0.00	0.4	
A-1	Shizuoka	12.6	39.1	48.3	37.9	51	94	4.09	8.20	19.7	Andosol
A-2	Kanto region	17.2	58.7	24.0	33.1	65	78	5.65	5.70	18.9	
A-3	Gunma	44.1	50.2	5.7	42.0	83	86	5.60	6.46	17.4	
A-4	Nagano	16.9	51.7	31.4	27.8	85	84	5.54	6.44	16.1	
B-1	Aichi	42.6	20.6	36.8	7.6	40	48	4.06	0.52	5.9	Brown forest soil
B-2	Fukushima	17.2	25.7	57.1	13.2	55	55	4.70	1.20	9.5	
B-3	Aichi	41.6	30.3	28.1	10.5	53	50	5.54	0.34	3.9	
C-1	Shiga	42.7	21.2	36.1	17.7	34	54	5.43	1.81	5.9	Cohesive soil
C-2	Saitama	8.6	50.4	41.1	15.5	55	52	6.10	0.70	5.6	
C-3	Nagano	40.9	39.8	19.3	10.9	62	60	5.66	2.70	6.8	
C-4	Aichi	6.8	54.2	39.0	32.2	81	81	5.54	4.22	12.4	
O-1	(OECD soil)	70.0	15.2	14.8	8.7	–	55	5.90	6.85	6.6	Cohesive soil

### Preparation of pseudo Cd-contaminated soils

CdCl_2_ solution was added to the soil samples at 1.5, 15, 150, or 1000 mg-Cd kg^–1^ dry soil, respectively. Hereinafter [Cd_Add_] (mg-Cd kg^–1^) refers to the Cd concentration for these artificially contaminated soils. The soil moisture content was adjusted to 50% WHC with distilled water, and 500 g of sample was transferred into a press-seal bag using a medicine spoon. As determined in preliminary experiments, the soil was mixed for 15 min in the bag and held for 7 days before experiments for the concentration of Cd in samples to equilibrate. We evaluated the aging effect by holding the Cd-added soils for 1, 3, 14, 42, and 133 days, respectively, and the chemical analyses and DGT analyses shown below were applied to those aged soil samples. The Cd concentrations in all sample soils were analyzed by ICP-MS (Agilent, 7500cx).

### DGT measurements and calculations

We used the DGT method to determine the bioavailable fraction of Cd^2+^ ions in water and soil [37]. The method uses DGT samplers (DGT Research Ltd., Lancaster, UK), which are plastic disks covered with a thin resin gel film (Chelex) that permits the entry of inorganic ions at a large diffusion velocity [12]. As specified in the manufacturer’s manual, the soil samples were moistened to 100% WHC and equilibrated for 24 h. A DGT sampler was stuck in a 100-g (dry weight) soil sample and held at 25°C for 24 h. All experiments were carried out twice. The resin gel film was then removed and immersed in 1 mL of 1-M nitric acid for 24 h. The time-averaged DGT-measured Cd ([Cd_DGT_], μg L^–1^) was calculated as

$$\left[Cd_{DGT}\right]=\frac{M_{DGT}\times \Delta g}{D\times t}$$

where *M*_DGT_ is the amount of metal accumulated in the resin per unit area (μg cm^–2^), Δ*g* is the total thickness of the diffusive gel layer (0.082 cm) and the filter membrane (0.014 cm), *D* is the diffusion coefficient of Cd in the diffusive gel (6.09 × 10^−6^ cm^2^ s^–1^), and *t* is the deployment time (86400 s). *M*_DGT_ was calculated as

$$M_{DGT}=C_{Cd}\times \frac{V_{HNO3}+V_{g}}{A\times f_{e}}$$

where *C*_Cd_ is the measured [Cd] in the 1-M HNO_3_ (μg L^–1^), *V*_HNO3_ is the volume of HNO_3_ used to elute the resin gel film (1 mL), *V*_gel_ is the volume of the film (0.16 mL), *A* is the exposure area of the filter membrane to elute (3.14 cm^2^), and *f*_e_ is the elution factor for each metal (0.8). Some previous studies have performed a correction method of [Cd_DGT_] for soil considering a soil-specific constant, R_diff_ [12,38]; however, we did not correct [Cd_DGT_] by R_diff_. This was because we could not obtain a rational R_diff_ value due to the concentration dependency of the distribution coefficient (K_d_) [31], which is a key parameter to calculate R_diff_ and is postulated as a constant for calculations of R_diff_.

We used the measured data of day 7 due to data availability. The differences between [Cd_DGT_] of day 7 and those of the other days (days 1, 3, 14, 42, and 133) were small (See **S1 Fig**; details are discussed in the Results section).

### Statistical analyses for characterizing soil properties; construction of linear regression model

We examined the effects of soil properties on [Cd_DGT_] in three steps. First, we conducted a factor analysis (FA) to choose some major influential physico-chemical parameters that describe physicochemical differences among the soils (thereafter, we named them as representative physicochemical parameters). The maximum likelihood procedure was used, followed by the promax rotation. Factor scores were estimated using the regression method. Second, we examined the effects of the representative physicochemical parameters of soils on [Cd_DGT_] by multiple regression analysis. Finally, we developed a multiple regression model that estimates [Cd_DGT_] from [Cd_Add_], CEC, and pH. All statistical analyses were conducted in R software [39]. All data are presented in the Supplementary Material.

### Interpretation by biotic ligand model

For evaluation of the results of the linear regression model, we obtained the biotic ligand model parameters as described.

To measure cations (Na, Ca and Mg) in soil solutions of artificially contaminated soil samples, we added water at 200% of WHC; for example, for S-1, we added (0.232 × 2) L of water per kg of soil (dry weight). Concentrations of the cations were measured on a 200% WHC basis. To convert them to a 100% WHC basis, we doubled them. This assumption is valid when concentrations are far below saturation. Proton concentrations were calculated as [H^+^] = 2 × 10^–pH^, in which the factor of 2 gave [H^+^] on a 100% WHC basis. The concentrations of cations in solution were determined by ICP-MS (Agilent, 7500cx).

To convert added Cd concentration ([Cd_Add_]. Unit: [mg/kg]) into Cd concentration in sample ([Cd_Add_-s]), we assumed that all the added CdCl_2_ dissolved; thus, for example, for S-1 with 1000 mg kg^–1^, [Cd_Add_-s] = 1000 mg / (0.232 mL × 2) = 2155 mg L^–1^. All concentrations were converted into molar units. We used the concentration of the sample on day 3 due to data availability. Differences between the concentrations for Ca, Mg and H on day 3 and those on the other days, i.e. days 1, 7, 14, 42, and 133, were small.

The concept for the biotic ligand model is based on previous researches [40–42]. For the sake of simplicity, a single type ligand in a soil was assumed, considering the kinetics of Cd, Ca, Mg and H in our model. The type of ligand in each soil type was assumed to be different; hence, model parameters were estimated separately in each soil. A simple mass-action type model was assumed for the kinetics, such that

[M]+[L]↔[ML] (1)

where M is the cation (Cd, Ca, Mg and H) and L is the ligand, and [ML] is the cation-ligand complex.

The underlying scenario in the mathematical analysis is that cations other than Cd are at equilibrium, and the addition of Cd disturbs the kinetico-dynamics and shifts the equilibrium. At the new equilibrium, we measured cation concentrations in solution. Those cations are exchanged from the cation-ligand complex [ML].

Let us denote the stability constants between [M]+[L] and [ML] at equilibrium by *K*_M_, then there is the relationship:

$$K_{M}=\frac{\left[ML\right]}{\left[M][L\right]}$$ (2)

The concentration of the free ligand ([L]) at equilibrium is common for Cd and the various cations. Using the relationship, we obtain, for example,

$$[L]=\frac{\left[CdL\right]}{K_{Cd}[Cd]}=\frac{\left[CaL\right]}{K_{Ca}[Ca]}.$$ (3)

This is an example between Cd and calcium, but would also hold true for other cations.

Considering the mass balance, we have

$${\left[M\right]}^{0}=[M]+[ML].$$ (4)

where [M]^0^ is the total concentration of metal and cations. Because the concentrations of cations were analyzed, [M] for Cd and cations were available. The total concentration of Cd was known, whereas that of each cation not. In addition to estimating affinity constants, the total concentrations of cations other than Cd were also estimated.

By modifying Eq 3 with a combination of Eq 4, we have

$$\frac{1}{\left[Ca\right]}=\frac{1}{{\left[Ca\right]}^{0}}(1+\frac{K_{Ca}}{K_{Cd}}\frac{{\left[Cd\right]}^{0}-[Cd]}{\left[Cd\right]}).$$ (5)

By considering that 1/[Ca] is a dependent variable while ([Cd]^0^ - [Cd])/[Cd] is an independent variable, linear regression analysis could be conducted and parameters could be estimated with 1/[Ca]^0^ as the y-intercept and K_Ca_/(K_Cd_ [Ca]^0^) as the slope.

Another equation which is true at an equilibrium is

$$[CdL]=\frac{K_{Cd}[Cd]{\left[L\right]}^{0}}{{1+K}_{Cd}[Cd]{+K}_{Ca}[Ca]{+K}_{Mg}[Mg]{+2K}_{H}[H]}.$$ (6)

A “2” is required in front of *K*_H_ because protons are monovalent and the equation can be arranged to be

$$[CdL]=\frac{[Cd]{\left[L\right]}^{0}}{\frac{1}{K_{Cd}}[Cd]+\frac{K_{Ca}}{K_{Cd}}[Ca]+\frac{K_{Mg}}{K_{Cd}}[Mg]+2\frac{K_{H}}{K_{Cd}}[H]}.$$ (7)

The left-hand-side (LHS) of Eq 7 ([CdL] = [Cd]^0^ - [Cd]) can be estimated from the observed data; here, [CdL] was assumed to be the difference of added Cd from free-ion Cd. The initial Cd ([Cd]^0^) was assumed to be equal to the added Cd. Affinity constants of the cation divided by that of Cd in right-hand-side (RHS) were already known from the previous regression analysis. The only unknown parameters were the total concentrations of ligand ([L]^0^) and *K*_Cd_. We first computed a squared sum of differences between LHS and RHS, and then the parameter values which minimized the squared sum were determined.

## Results

### Bioavailability measured by DGT

**Fig 1:**
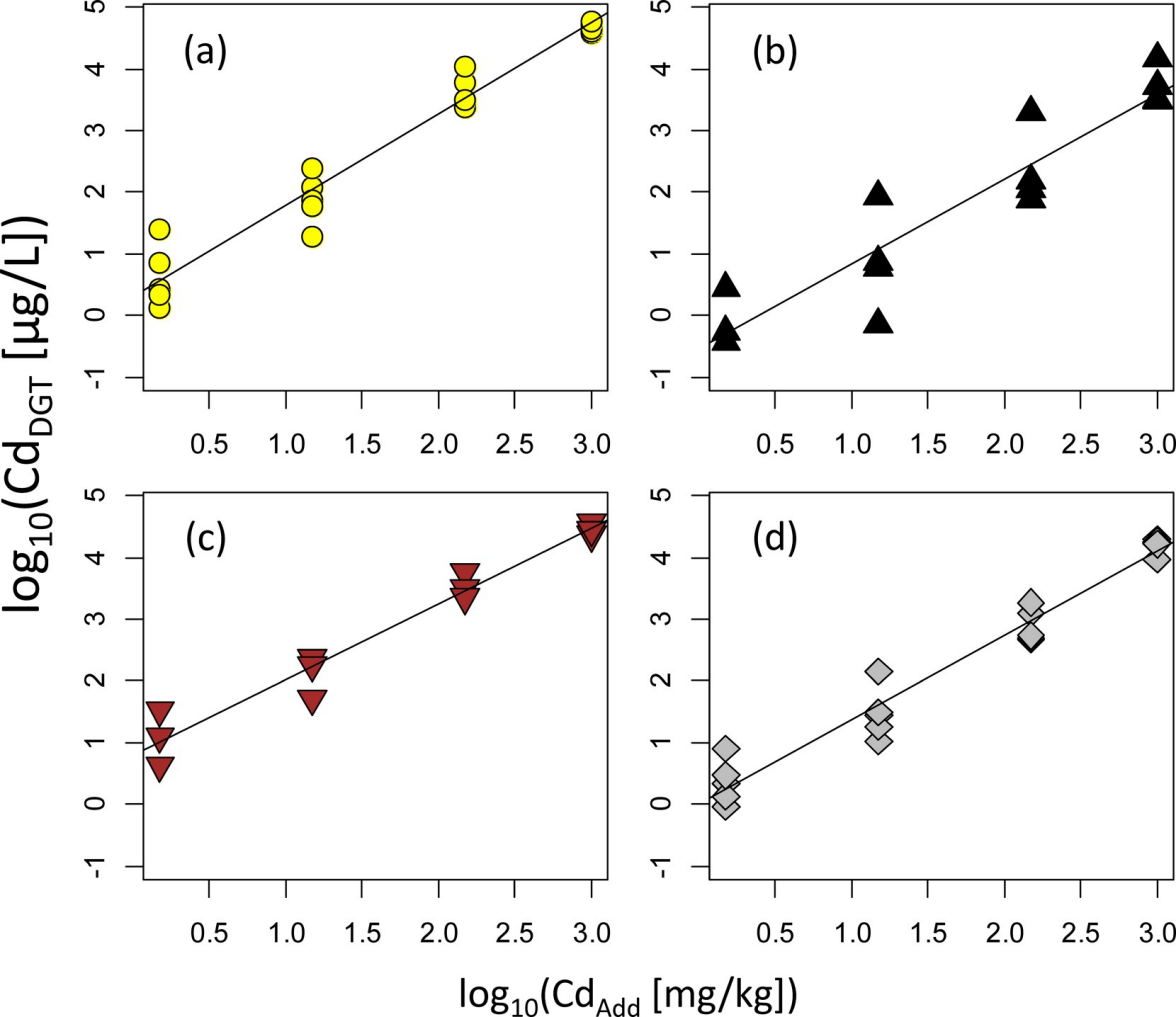
Relationship between [Cd_Add_] and [Cd_DGT_] for (a) sandy soils, (b) andosols, (c) brown forest soils, and (d) cohesive soils.

We measured [Cd_DGT_] for 17 samples ([Cd_DGT_] data were shown in S1 Table). In each soil type, log_10_[Cd_Add_] and log_10_[Cd_DGT_] showed a linear relationship (Fig 1). We measured [Cd_DGT_] for the soils aged for 1, 3, 7, 14, 42, and 133 days (S1 Fig).

As for [Cd_Add_] of 15, 150 and 1000 mg/kg, time-course changes in concentrations were analyzed by a three-way analysis of variance (ANOVA) to test the importance of the influence of aging. The influence of aging was insignificant (S1 Fig; p < 0.05), suggesting that aging had a minimal role in our samples. Thus, we assumed the samples reached equilibrium on day 1 and thereafter there were no significant concentration changes. We used the data of day 7 for further analyses because the cation data were available on day 7.

### Identification of important soil properties

#### Correlations among soil properties

**Fig 2:**
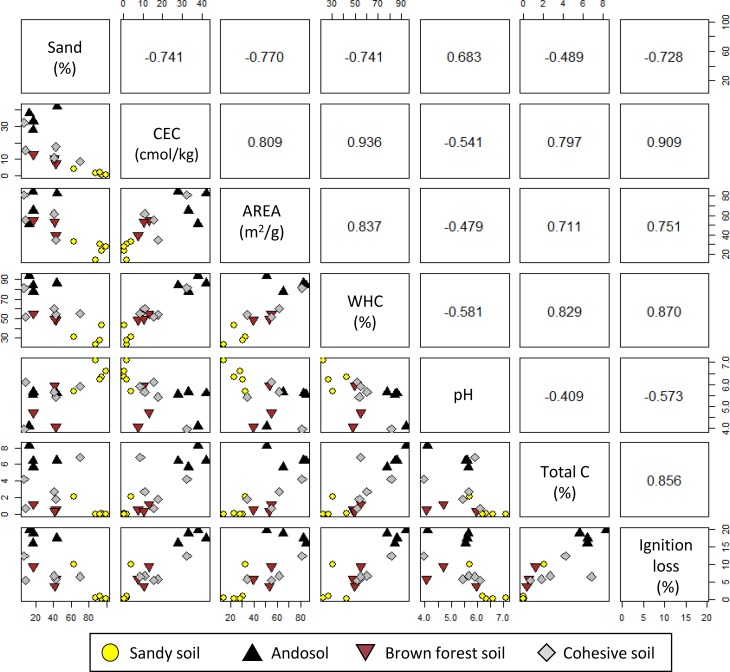
Correlation plots of physicochemical properties of 17 soil samples. Lower left, correlation plots; upper right, correlation coefficients. WHC, water-holding capacity.

**Fig 3:**
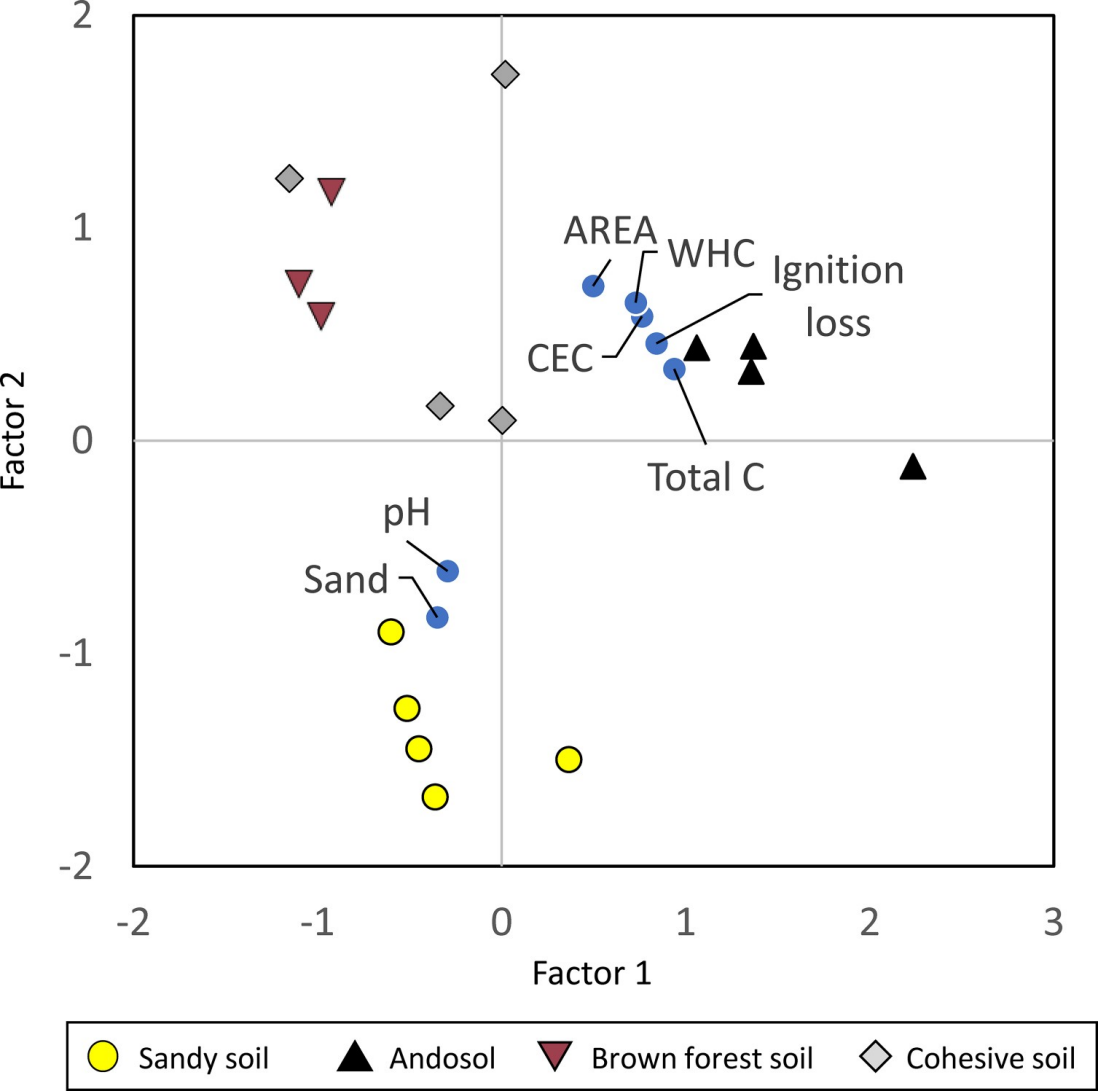
Plot of factor analysis scores for soil properties and 17 soil samples.

Among soil parameters, CEC had a relatively high correlation with specific surface area, WHC, total C, and ignition loss (r > 0.8; Fig 2). Values of pH had a relatively high correlation with particle size (sand fraction; r = 0.7). Because the interdependency of multiple soil properties leads to the problem of multicollinearity in multiple regression analysis, we performed factor analysis (FA) to select representative factors that could explain the changes in [Cd_DGT_] measured in different soils. The results of FA showed that individual soil types formed two distinct groups (Fig 3), indicating that soil properties vary with soil type. Factor 1 explained 47% of the total variation in the data, and the cumulative contribution of Factor 1 and Factor 2 was 83%. These results suggest that the two factors explained most of the variance in the samples. As is also indicated by the correlation analysis, CEC, specific surface area, WHC, total C, and ignition loss were plotted near each other in Fig 3, and pH was plotted close to the sand fraction.

Based on the results of FA, we considered that either CEC or WHC can be used as a proxy variable for the first group. We selected CEC because it has been more commonly used to explain bioavailability than WHC [21,43,44]. We also selected pH as a proxy variable for the second group. Thus, in the following analysis of the effects of soil properties on [Cd_DGT_], we used CEC and pH as explanatory variables in multiple regression models.

#### Effects of CEC and pH on [Cd_DGT_]

**Fig 4:**
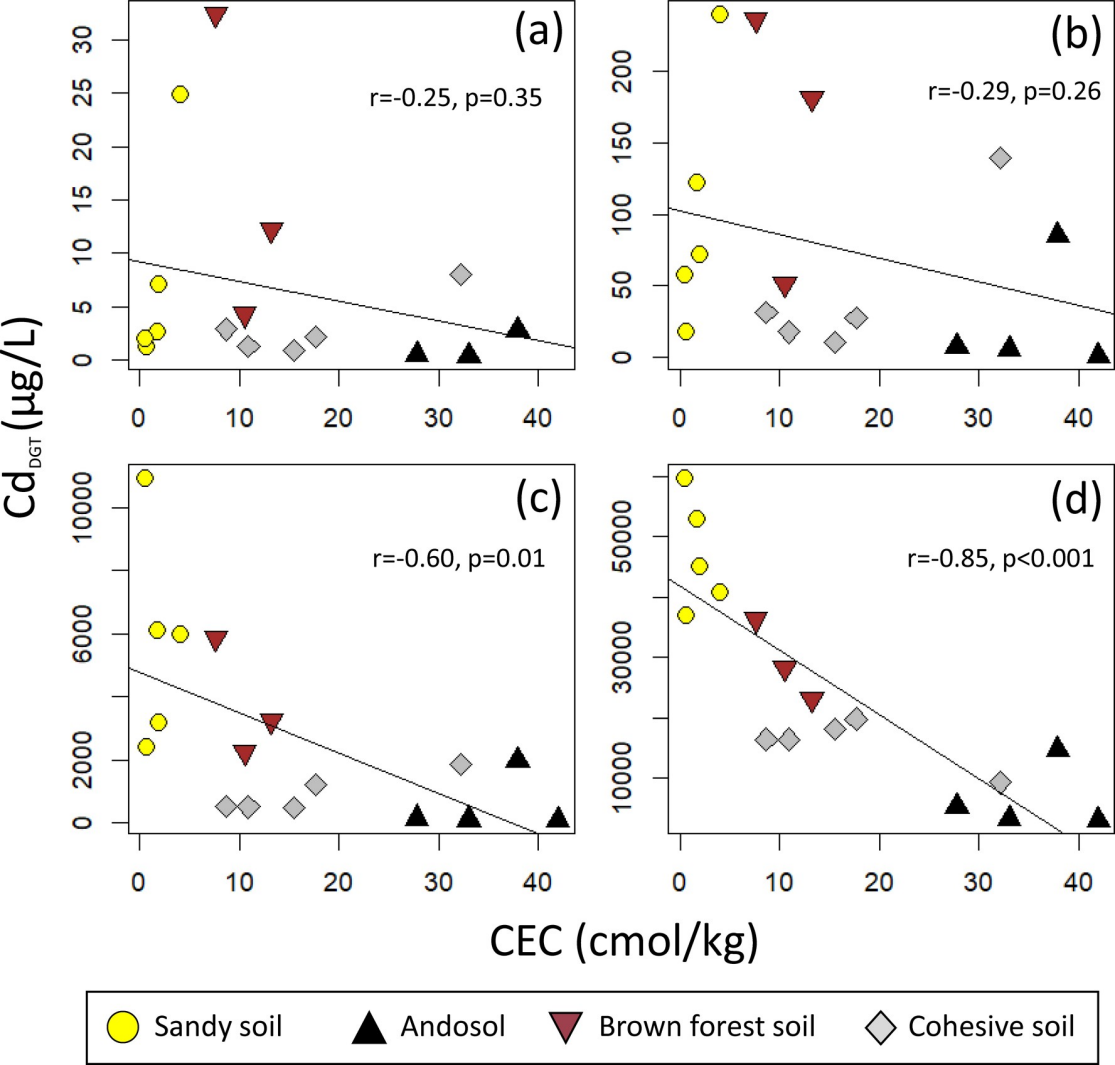
Relationship between CEC and [Cd_DGT_] when [Cd_Add_] = (a) 1.5, (b) 15, (c) 150, and (d) 1000 mg kg^–1^.

**Fig 5:**
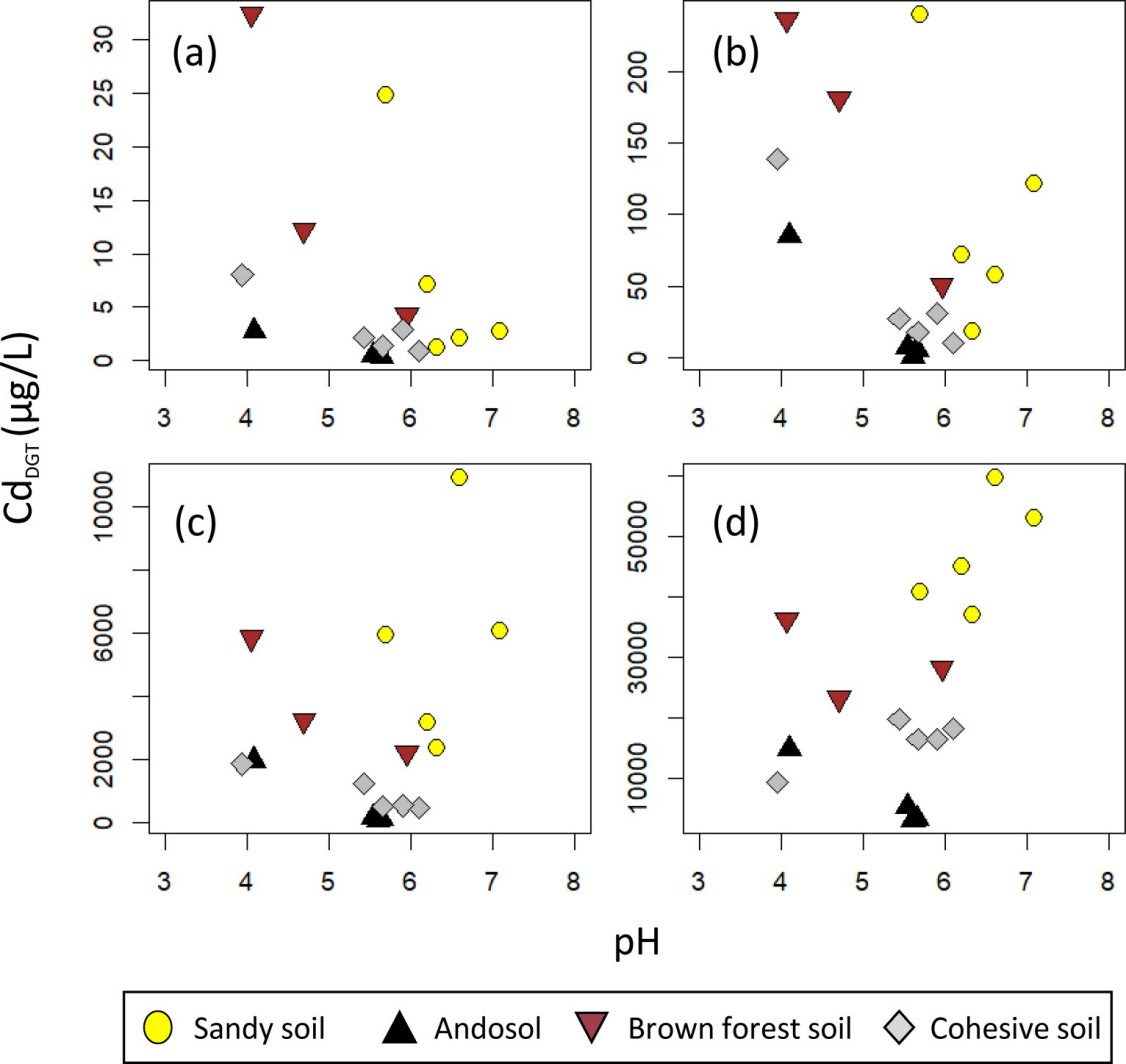
Relationship between pH and [Cd_DGT_] when [Cd_Add_] = (a) 1.5, (b) 15, (c) 150, and (d) 1000 mg kg^–1^.

We used CEC and pH to investigate the effect of soil properties on [Cd_DGT_]. [Cd_DGT_] tended to decrease as CEC increased (Fig 4). Also, [Cd_DGT_] generally decreased as pH increased within individual soil types when [Cd_Add_] was low (Fig 5A and 5B), but increased when [Cd_Add_] was high (Fig 5C and 5D). These apparently contradictory effects are likely due to the correlation between CEC and pH (e.g., sandy soil tended to have high pH and low CEC). To estimate the effect of CEC and pH separately, we used the following multiple regression analysis for data of each [Cd_Add_]:

$$log_{10}\left[Cd_{DGT}\right]=b_{1}\times CEC+b_{2}\times pH+intercept.$$ (8)

**Fig 6:**
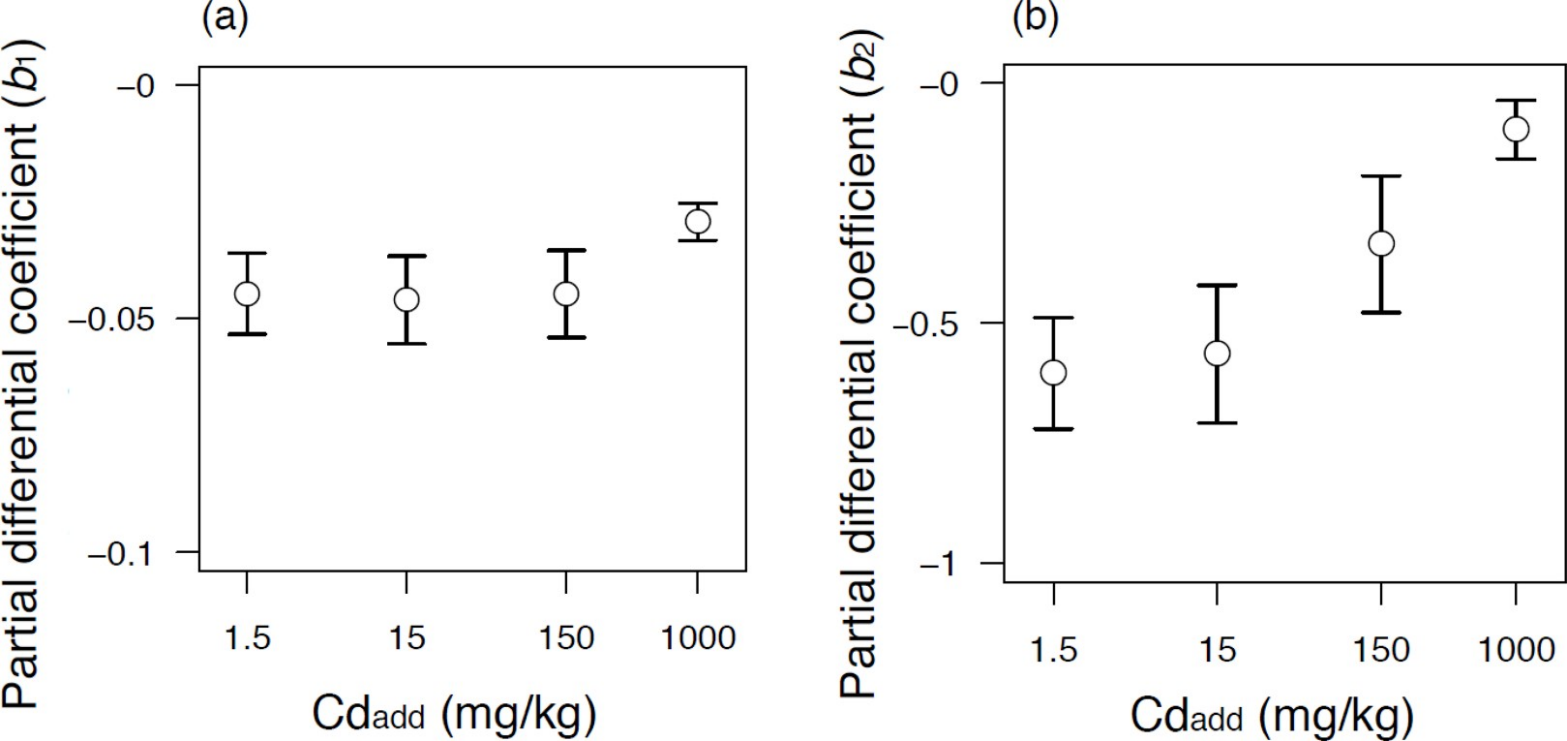
**Partial differential coefficients of (a) CEC and (b) pH for different values of [Cd**_**Add**_**] in** **Eq** 8. Error bars represent standard error of the estimates (N = 17).

CEC showed a significant effect regardless of [Cd_Add_] (i.e., *b*_1_ was relatively stable over the range of [Cd_Add_]; Fig 6(A). When [Cd_Add_] = 1.5 mg kg^–1^, *b*_1_ = –0.046. This means that [Cd_DGT_] decreases by 0.9× (= 10^−0.046^) as CEC increases by 1 cmol kg^–1^ when pH and [Cd_Add_] remain the same. The CEC of the soils used in this study ranged from 0.5 to 42 cmol kg^–1^, an 84× difference due to soil type. The decrease of [Cd_DGT_] with the increase of CEC is probably caused by an increase of Cd absorption capability on the surface of the soil particles.

In contrast, *b*_2_, the regression coefficient for pH, almost approached zero as [Cd_Add_] increased (Fig 6B). This indicated that the influence of pH on [Cd_DGT_] was reduced as more Cd was added. The value of *b*_2_ was –0.56 when [Cd_Add_] = 1.5 mg kg^–1^. This means that [Cd_DGT_] decreases by 0.27× (= 10^−0.56^) as the pH differs by +1 when CEC and [Cd_Add_] remain the same. This tendency is consistent with the results of Muhammad et al. (2012) demonstrating that a higher soil pH gave lower [Cd_DGT_]. However, we could not explain the phenomenon that occurred after equilibrium was attained by the intact pH of these soils only. We focused on pH and the other cations’ interaction by constructing a biotic ligand model in Section 3.4.

Note that the comparison of the values of the partial differential coefficients requires some care because the values depend on the units. When [Cd_Add_] = 1.5 mg kg^–1^, *b*_1_ = –0.62 and *b*_2_ = –0.49. This means that [Cd_DGT_] changes by 0.24× (= 10^−0.62^) with an increase of 1 SD of CEC, and by 0.32× (= 10^−0.49^) with a difference of 1 SD of pH.

### Construction of a statistical model to estimate [Cd_DGT_] from [Cd_Add_], CEC, and pH

We developed a linear regression model to estimate [Cd_DGT_] in all the experiments based on the [Cd_Add_], CEC of intact soil, and pH of intact soil. The model adds [Cd_Add_] to Eq (8) as an explanatory variable, and includes the interaction of pH × log_10_[Cd_Add_], because the effect of pH depended on [Cd_Add_]:

$$log_{10}\left[Cd_{DGT}\right]=b_{1}\times CEC+b_{2}\times pH+b_{3}\times log_{10}\left[Cd_{Add}\right]+b_{4}\left(pH\times log_{10}\left[Cd_{Add}\right]\right)+intercept$$ (9)

The result of multiple regression analysis using this model (Table 2) showed that all explanatory variables were significant (*P* < 0.05).

**Table 2:** Result of multiple regression analysis.

	Estimate *b*	Std. Error	t value	
(Intercept)	4.364	0.546	7.987	***
CEC	-0.041	0.004	-10.619	***
pH	-0.655	0.093	-7.013	***
log_10_[Cd_Add_]	0.562	0.253	2.217	*
pH×log_10_[Cd_Add_]	0.147	0.045	3.274	**

* Significant at 5% level

** Significant at 1% level

*** Significant at 0.1% level

### Constructing a biotic ligand model and validation of the model

In order to construct a biotic ligand model for these soils, first we obtained data of cation (i.e., Ca, Mg and H) concentrations in the soil solutions at equilibrium 3 days after Cd addition (Table 3). Higher cation concentrations were observed in soil with more [Cd_Add_]. This might be because high [Cd_Add_] brought out competition of receptor sites of cations that had existed in intact soil.

**Table 3:** Cation concentrations (mM and μM) at equilibrium (day 3) for artificially contaminated soils S-1, A-1, B-1 and C-1.

Sample No (Soil type)	[Cd_Add_] (mg/kg)	[Cd_Add_-s] (mM)	[Cd_DGT_] (μM)	Ca^2+^ (mM)	Mg^2+^ (mM)	H^+^ (mM)
S-1 (Sandy soil)	0	0.0	0.0	0.0125	0.00863	0.0000224
	15	0.29	0.031	0.0125	0.00982	0.0000283
	150	2.9	12	0.0629	0.0491	0.000356
	1000	19	730	0.249	0.179	0.00126
A-1 (Andosol)	0	0.00	0.00	0.00691	0.0103	0.100
	15	0.071	0.38	0.00704	0.0107	0.178
	150	0.71	9.4	0.0125	0.0168	0.283
	1000	4.7	210	0.0274	0.0256	0.502
B-1 (Brown Forest Soil)	0	0.00	0.00	0.0599	0.0741	0.0448
	15	0.14	1.0	0.0724	0.103	0.0564
	150	1.4	31	0.210	0.288	0.142
	1000	9.2	480	0.414	0.535	0.200
C-1 (Cohesive soil)	0	0.00	0.00	0.773	0.272	0.000893
	15	0.12	0.043	0.724	0.272	0.00159
	150	1.2	1.5	1.92	0.428	0.00178
	1000	8.3	110	11.0	2.14	0.0252

**Fig 7:**
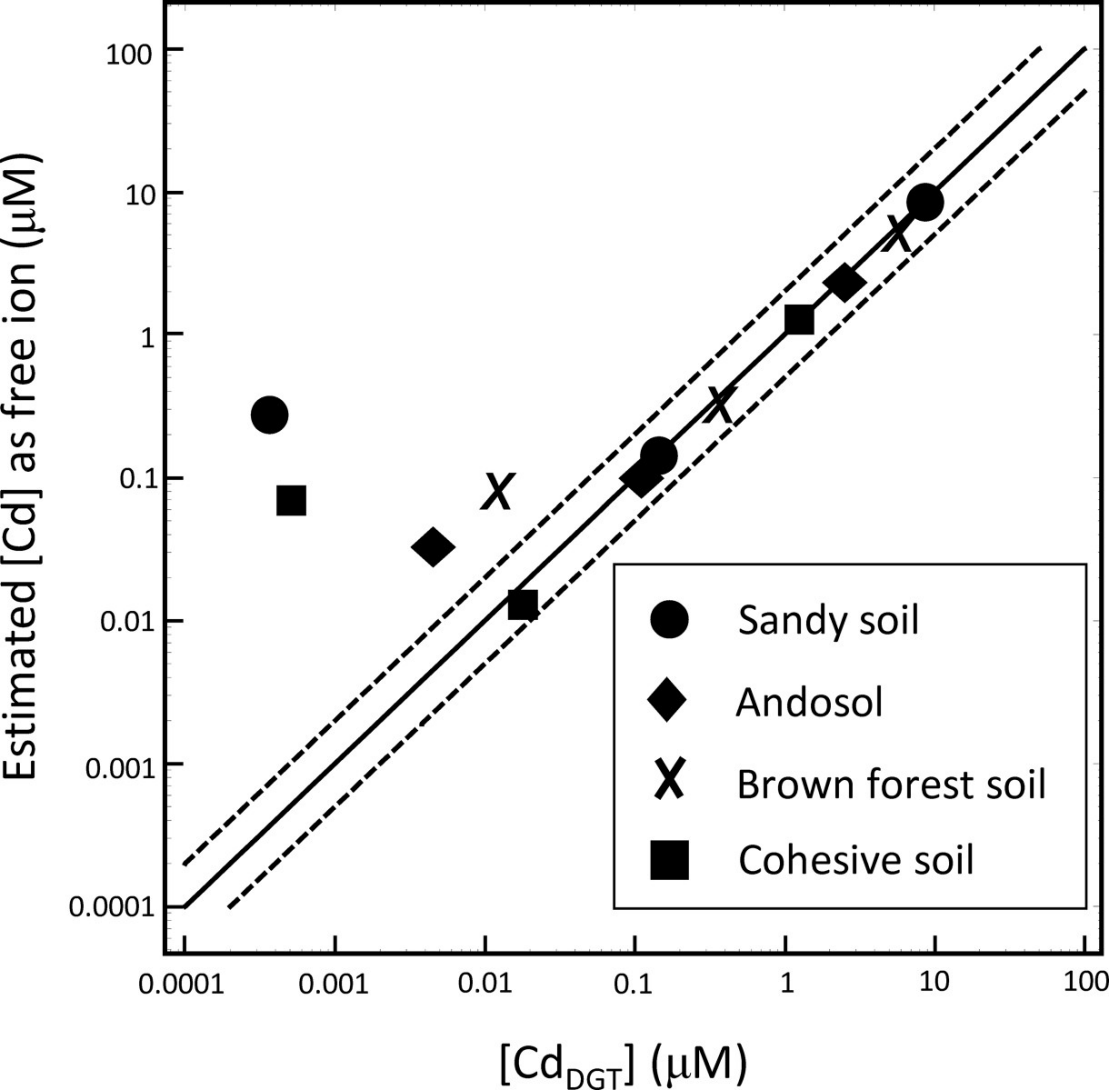
Comparison of observed [Cd] as [Cd_DGT_] and estimated free [Cd] for samples S-1, A-1, B-1 and C-1 by the ligand model. Dashed lines show a factor of 2.

We estimated the total concentrations of Ca, Mg, H and the ligand, and affinity constants for the biotic ligand model (Table 4). The total ligand concentration ([L]^0^) in S-1 (sandy soil) was estimated to be higher than those in other soils. This result is counterintuitive because typically, sandy soil has a characteristic of weak absorption [31,45]. The relationship between observed ([Cd_DGT_]) and estimated (free [Cd]) Cd concentrations is shown in Fig 7. The result showed that model-estimated concentrations were within plus or minus a factor of 2 of observed concentrations in the higher concentration range (i.e., > 0.01). At the lower concentration range (<0.01 μM), the estimated values were 10 to 100 times higher than those observed; the reason for the discrepancy is unclear. The other cations had a similar tendency (S2(A)–S2(C) Fig).

**Table 4:** Estimated total concentrations of Ca, Mg, H and ligand (L) (mM) for artificially contaminated soils and affinity constants of biotic ligand model (M^-1^).

Sample No (Soil type)	Estimated total concentration (mM)				Affinity constant (M^-1^)			
	[Ca]^0^	[Mg]^0^	[H]^0^	[L]^0^	K_Cd_	K_Ca_	K_Mg_	K_H_
S-1 (Sandy soil)	2.24	1.04	0.00037	11.14	2387.81	24.48	23.66	38.71
A-1 (Andosol)	0.59	0.33	0.26	2.61	3645.81	806.31	387.39	240.87
B-1 (Brown Forest Soil)	0.31	0.38	0.13	4.32	1006.38	716.13	609.57	170.98
C-1 (Cohesive soil)	4.95	0.58	0.0026	9.08	16634.4	876.66	236.53	88.13

## Discussion

### Multiple regression model

We constructed a multiple regression model to estimate Cd bioavailability that was generally applicable to representative Japanese soils. This model will be useful in assessing the potential vulnerability of Japanese soils to Cd.

We used intact soil pH, intact CEC and added Cd concentration as explanatory parameters, and [Cd_DGT_] as an indicator of bioavailability. For ecological risk assessment of soil, it is important to take the metal bioavailability into account in addition to measuring the total concentrations of metals. Integrating parameters and expressing soil characteristics using a few representative parameters is important for constructing an applicable model even though many soil parameters are available. In this study, we integrated parameters as independent parameters using FA. We observed that the influence of pH on [Cd_DGT_] was reduced as more Cd was added. This might be because added Cd had been purged of H+ or other cations. This was an interesting interaction among cations. The biotic ligand model confirmed that the selected independent parameters had significant explanatory power.

### Biotic ligand model

In order to explain the above phenomenon, we developed the biotic ligand model. Irrespective of the simplifications and approximations of the model, the predicted concentrations in all soils were reasonable.

Most studies so far (e.g., Ruello et al. 2008; Stephan et al. 2008) have attempted to describe the link between environmental concentrations of metals and internal concentrations of metals in biota as a function of physico-chemical properties in soils. There are at least two processes in the behavior of metals in passing from the environment to biota. In the first process, metals bind to ligands in soils; metals that do not bind to the ligand exist as free ions and become available for uptake by organisms. It is very important to consider these two processes separately to understand the toxic effect of a metal mixture [46]. The biotic ligand model is good at expressing these two processes.

The results brought us a speculation that high [Cd_Add_] brought out competition of receptor sites of cations that had existed in intact soil. Such cations were exchanged (or purged) from receptor sites. Generally, that would lead to a decline of pH. The biotic ligand model constructed here mimicked this mechanism well. As a previous study [47] pointed out, the adsorption of Cd often correlates with the CEC of the soil at high solution concentrations of Cd (>10 mg/L). During cation exchange, Cd generally exchanges with adsorbed calcium and magnesium because the ionic radius of Cd^2+^ is comparable to that of Ca^2+^ and, to a lesser extent, Mg^2+^ [47].

The biotic ligand model we constructed has some limitations. There is some discrepancy in estimated values and measured values at the low (<0.1μM = 0.005 mg-Cd/L) range of concentration (Fig 7). There are a couple of possibilities to explain this discrepancy. One is that [Cd_Add_] is too high and the model was fit to optimize the high-concentration region; hence, the model is less predictable in the low-concentration region. Another possibility is that in our analysis a mass balance between added Cd and total amount of exchanged cation is assumed. The absorbed Cd (i.e., the difference of added Cd and Cd exchanged into solution), however, exceeded the total amount of exchanged cations; hence, this assumption may be improper in some soils. This suggests that there may other cations in addition to those assumed (Ca, Mg and H) in the present study; this may also be the reason why the estimated total carbon in sandy soil is much higher than that in other soils (see Table 4). More detailed analysis, particularly in sandy soil, is necessary.

Although we examined the correlation between pH and [Cd_DGT_] in a variety of soils, we did not closely examine the effect of pH on [Cd_DGT_] within a soil type or the effect of CEC on [Cd_DGT_] in soils of the same pH. Future studies will be needed to test these effects.

## Conclusion

We measured selected properties of 17 samples comprising 4 Japanese soil types and used the DGT method to leach out added Cd. We statistically analyzed correlations between soil properties and Cd bioavailability. Based on the results, we developed a statistical model to estimate [Cd_DGT_] from [Cd_Add_], CEC, and pH. The correlation between CEC and bioavailability had a low dependence on [Cd_Add_], and bioavailability decreased with increasing CEC. In contrast, the correlation between pH and bioavailability had a high dependence on [Cd_Add_], and the effect of pH on [Cd_DGT_] became smaller as [Cd_Add_] increased. In addition, [Cd_DGT_] tended to increase with decreasing pH when [Cd_Add_] was low. Thus, we proposed a linear regression model which included the interaction of intact pH and [Cd_Add_]. This simplified but realistic model will be useful in estimating the vulnerability of four representative Japanese soils and facilitate the identification of higher-risk sites for soil ecosystems.

## Supporting information

S1 Fig [Cd_DGT_] at aging in (a) sandy soil (Sample: S-1), (b) Andosol (A-1), (c) Brown Forest Soil (B-1), and (d) cohesive soil (C-1).
(PDF)

S2 Fig **Comparison of observed and estimated (a) [Ca**^**2+**^**], (b) [Mg**^**2+**^**] and (c) [H**^**+**^**] for samples S-1, A-1, B-1 and C-1 by the ligand model.** Dashed lines show a factor of 2.
(PDF)

S1 Table Result of [Cd_DGT_] (μg/L) (day 7). (PDF)
