# Supplementary figures and images for: Model construction for estimating potential vulnerability of Japanese soils to cadmium pollution based on intact soil properties
Source: PLoS One. 2019 Jun 14;14(6):e0218377. doi: 10.1371/journal.pone.0218377 (PMC6570033; doi:10.1371/journal.pone.0218377)

S1 Fig

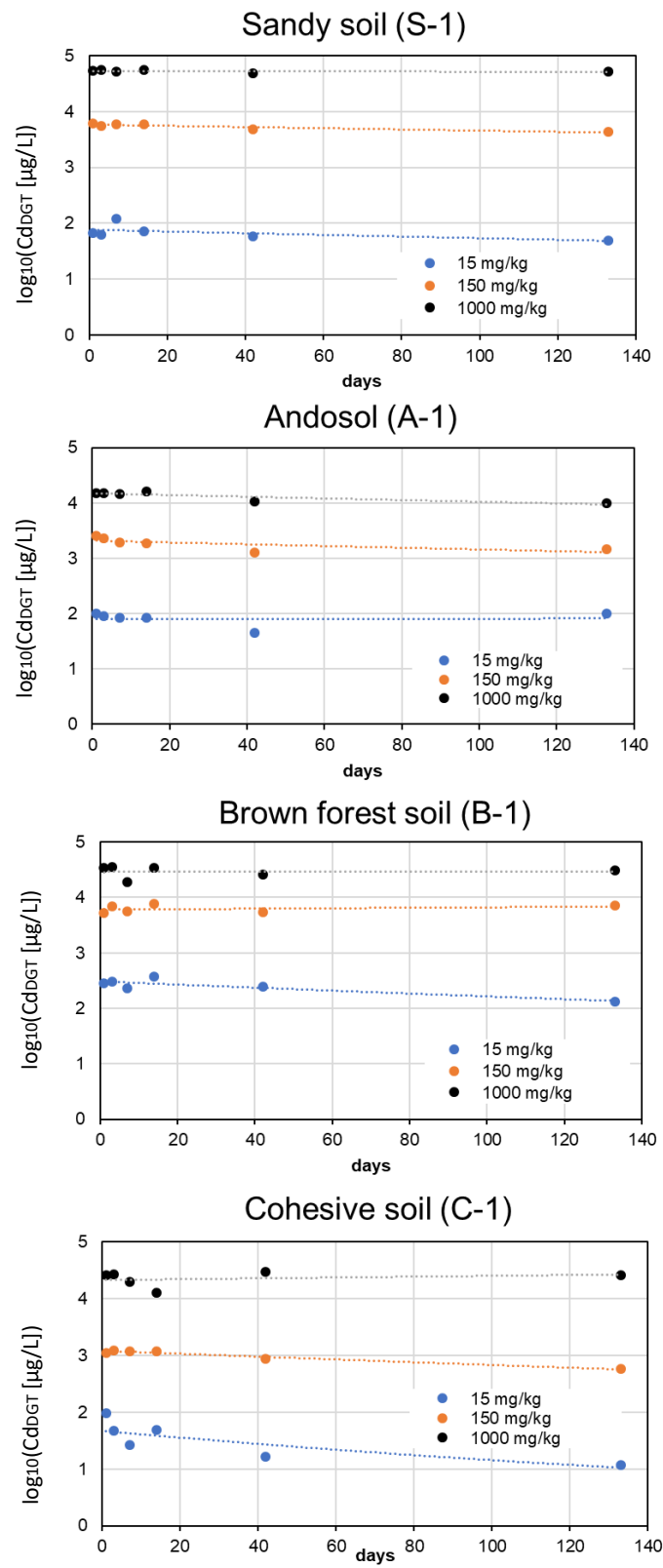

Supplement: S1 Fig — [CdDGT] at aging in (a) sandy soil (Sample: S-1), (b) Andosol (A-1), (c) Brown Forest Soil (B-1), and (d) cohesive soil (C-1). (PDF) [file pone.0218377.s001.pdf]

S2 Fig

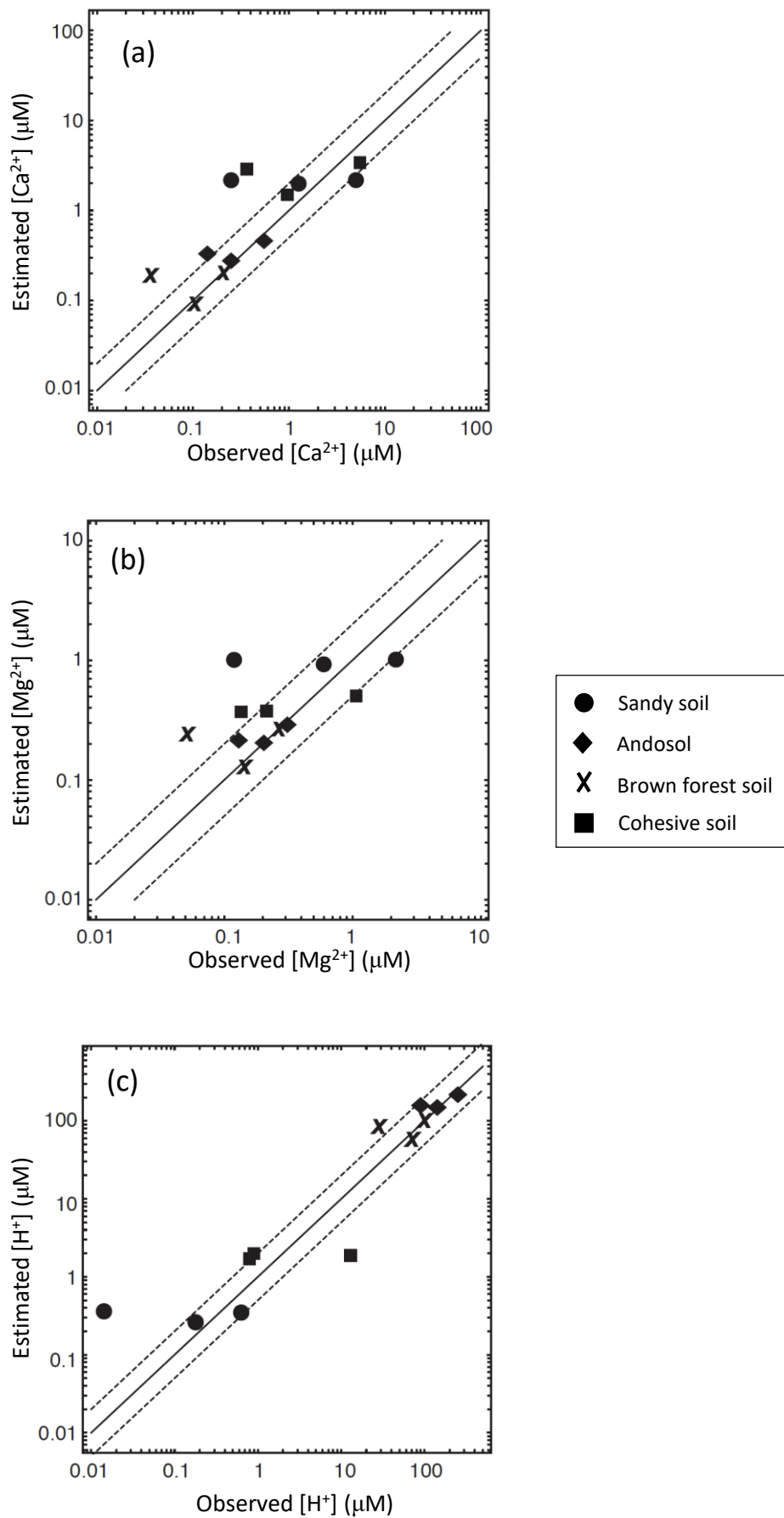

Supplement: S2 Fig — Comparison of observed and estimated (a) [Ca2+], (b) [Mg2+] and (c) [H+] for samples S-1, A-1, B-1 and C-1 by the ligand model. Dashed lines show a factor of 2. (PDF) [file pone.0218377.s002.pdf]
