# Supplementary material for: Model construction for estimating potential vulnerability of Japanese soils to cadmium pollution based on intact soil properties
Source: PLoS One. 2019 Jun 14;14(6):e0218377. doi: 10.1371/journal.pone.0218377 (PMC6570033; doi:10.1371/journal.pone.0218377)
Supplement: S1 Table — (PDF) [file pone.0218377.s003.pdf]

S1 Table. Result of [Cd_{DGT}] (μg/L) (day 7).

Soil type	Sample name	Sampling location	Latitude, Longitude	[Cd_{Add}]				
				0 mg/kg	1.5 mg/kg	5 mg/kg	150 mg/kg	1000 mg/kg
Sandy soil	S-1	Toyama	NA	< 0.25	3	122	6110	53100
	S-2	Shizuoka	NA	< 0.25	1	19	2400	37100
	S-3	Gifu	NA	< 0.25	25	240	5980	40900
	S-4	Nagano	NS	< 0.25	7	72	3200	45100
	S-5	Yamaguchi	NA	< 0.25	2	58	11000	59800
Andosol	A-1	Shizuoka	34.808291, 138.134117	0	3	86	1980	14700
	A-2	Kanto region	NA	< 0.25	0	6	114	3200
	A-3	Gunma	NA	< 0.25	< 0.25	1	78	2990
	A-4	Nagano	36.22719, 137.86041	< 0.25	1	7	156	5350
Brown Forest Soil	B-1	Aichi	34.896900,137.265087	1	32	236	5860	36200
	B-2	Fukushima	NS	1	12	181	3240	23200
	B-3	Aichi	34.893646,136.867716	< 0.25	4	51	2230	28200
Cohesive soil	C-1	Shiga	35.256133, 136.215849	0	2	27	1220	19800
	C-2	Saitama	NA	< 0.25	1	11	463	18100
	C-3	Nagano	36.20721, 137.86309	< 0.25	1	18	501	16400
	C-4	Aichi	34.872108,136.859516	1	8	139	1850	9270
OECD soil	O-1	–	NA	< 0.25	3	31	535	16400

NA: Geographical coordinates were unavailable because the sample was purchased from the market.

NS: Geographical coordinates were unavailable because the sample was complimentarily provided by relevant authorities for promotion of scientific research.
